# Acute presentation of cocoon abdomen as septic peritonitis mimicking with strangulated internal herniation: a case report

**DOI:** 10.1186/s40792-021-01179-7

**Published:** 2021-04-13

**Authors:** Sabah Uddin Saqib, Rimsha Farooq, Omair Saleem, Sarosh Moeen, Tabish Umer Chawla

**Affiliations:** Aga Khan University Hospital, Karachi, Pakistan

**Keywords:** Small bowel obstruction, Cocoon abdomen, Septic peritonitis, Emergency laparotomy

## Abstract

**Background:**

Abdominal cocoon syndrome is a rare cause of intestinal obstruction in which loops of small bowel get entrapped inside a fibro-collagenous membrane. Condition is also known in the literature as sclerosing peritonitis and in the majority of cases, it has no known cause. Although the majority of patients exhibit long-standing signs and symptoms of partial bowel obstruction in an out-patient clinic, its acute presentation in the emergency room with features of sepsis is extremely rare. This case report aims to describe the emergency presentation of cocoon abdomen with septic peritonitis.

**Case presentation:**

A 35-year-old male with no known co-morbidity and no prior history of prior laparotomy presented in emergency room first time with a 1-day history of generalized abdomen pain, vomiting, and absolute constipation. He was in grade III shock and had metabolic acidosis. The clinical impression was of the perforated appendix, but initial contrast-enhanced computed tomography (CECT) was suggestive of strangulated internal herniation of small bowel. Emergency laparotomy after resuscitation revealed hypoperfused, but viable loops of small bowel entrapped in the sclerosing membrane. Extensive adhesiolysis and removal of the membrane were performed and the entire bowel was straightened. Postoperatively he remained well and discharged as planned. Histopathology report confirms features of sclerosing peritonitis.

**Discussion:**

Cocoon abdomen is a very rare cause of acute small bowel obstruction presenting in an emergency with features of septic peritonitis. Condition is mostly chronic and generally mimics abdominal TB in endemic areas like India and Pakistan. A high index of suspicion is required in an emergency setting and exploratory laparotomy is diagnostic and therapeutic as well and the condition mimics internal herniation in acute cases.

**Conclusion:**

Cocoon abdomen as a cause of septic peritonitis is extremely rare and might be an unexpected finding at laparotomy. Removal of membrane and estimation of the viability of entrapped bowel loops is the treatment of choice, which may require resection in the extreme case of gangrene.

## Background

Intestinal obstruction with the risk of strangulation is a very common surgical emergency [1]. Many times there is a predisposing risk factor present such as the prior history of laparotomy or hernia. However, at times, unusual cases of bowel obstruction such as abdominal cocoon (AC) may present the first time in an emergency without prior significant medical history [2]. AC also is known as sclerosing peritonitis is generally regarded as a chronic condition, in which the small bowel is partially or encased by a thick fibro-collagenous membrane which gives a cocoon-like appearance [3]. Entrapment of bowel or twisting might lead to vascular insufficiency and a state of low perfusion in the bowel, which can present with signs of peritonitis and on rare occasions with systemic sepsis. In the emergency room, it may be picked up initially as perforated viscus or internal herniation and is not often suspected preoperatively. We report a case after written informed consent from the patient, as per SCARCE guidelines [4], in which abdomen cocoon syndrome was presented as frank peritonitis with systemic signs and its radiologic features mimicked internal herniation of bowel.

## Case presentation

A 35-year-old male with no known comorbidity presented first time in the emergency department with a 24-h history of generalized abdominal pain, which initially started around the umbilicus. He also complained of nausea, 6 episodes of bilious vomiting, and inability to move his bowel during this period. His past medical and surgical history was unremarkable and had never complained of similar symptoms ever before. He was a chronic smoker and was taking one pack per day for 10 years.

On presentation to the emergency room, he was anxious but alert. He looked extremely dehydrated with a heart rate of 132 beats per minute and blood pressures of 96/76 mm of hg. His focused abdominal examination showed generalized distention and tenderness with rebound tenderness in the right lower abdomen suggestive of focal peritonitis. The initial clinical assessment suggested that he is in hypovolemic shock showed with focal peritonitis in the right iliac fossa likely because of a perforated appendix.

**Fig. 1:**
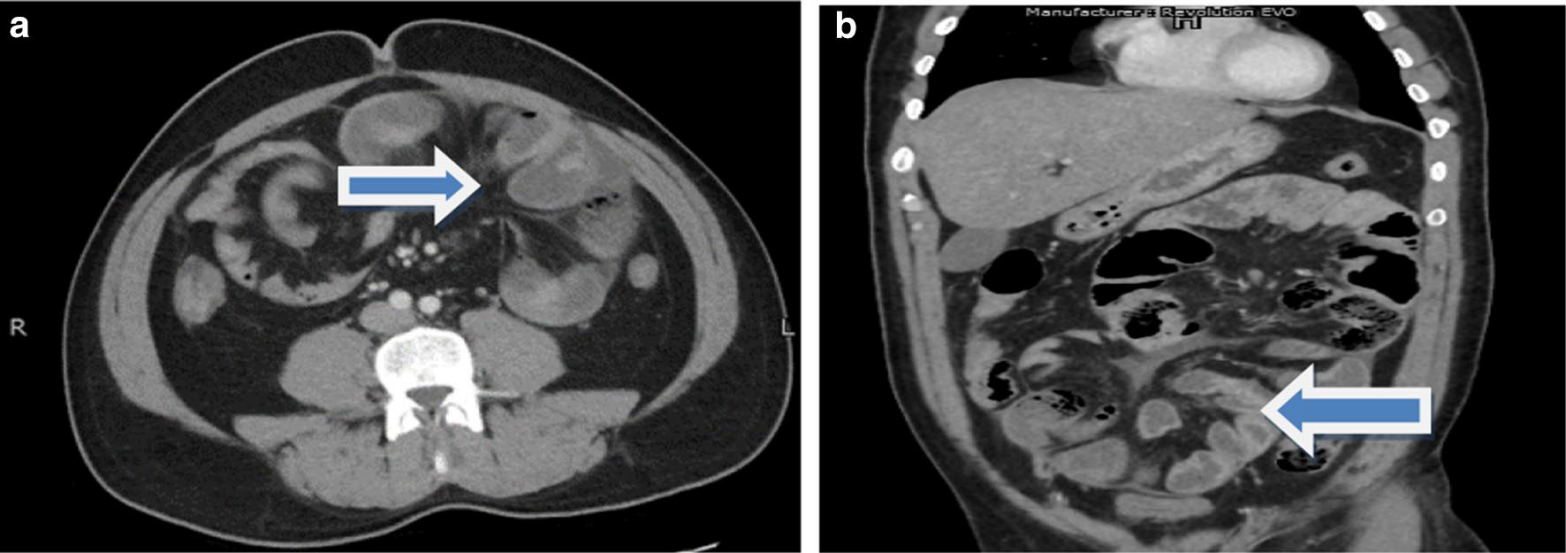
**a** and **b** Axial and coronal views of contrast-enhanced abdominal CT scan in which arrow shows twisted bowel loops raising the possibility of internal herniation

In the emergency room, he was resuscitated with intravenous fluids, antibiotics, antiemetics, and analgesics. Nasogastric tube and transurethral Foley’s catheter were passed. His initial serology workup showed blood urea nitrogen of 13 mg/dl and creatinine of 1.1 mg/dl and white blood cell (WBC) count of 22.4 per cubic millimeter and arterial lactic acid of 3.8 mmol/L. He responded to fluid resuscitation and his heart rate came down to 105 beats per minute. Contrast-enhanced CT (CECT) of the abdomen was performed which showed a cluster of small bowel loops in the lower abdomen and the pelvic region anteromedial to the mesocolon with a surrounding sac that appeared to protrude through the defect in the mesocolon on the right side of the midline. The small bowel loops distal to this herniated sac as well as the large bowel loops appeared collapsed. These findings raised the possibility of strangulated internal hernia with small bowel ischemia (Fig. 1a, b).

**Fig. 2:**
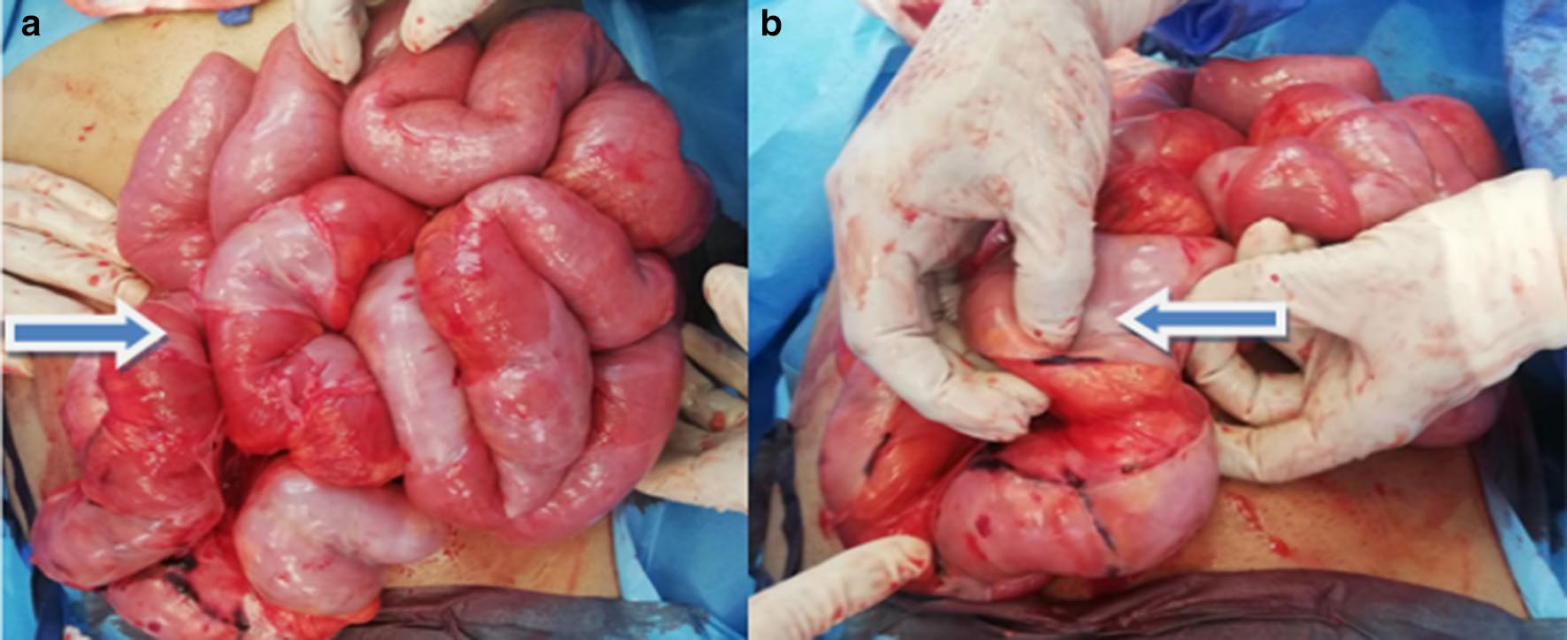
**a** Demonstrates clumped small bowel loops like a cocoon. **b** Shows sclerosing membrane

Based on the above findings he was planned for the exploratory laparotomy with the possibility of bowel resection and anastomosis or stoma. However, exploratory laparotomy revealed contrary findings as the entire small bowel was clumped together within a sclerosing membrane like a cocoon (Fig. 2a, b). Part of the small bowel loops was grossly dilated, but there was no zone of transition. The entire sclerosing membrane was removed with sharp dissection, which was resulting in obstruction. A small part of the small bowel measuring 30 cm near terminal ileum appeared to be hypoperfused initially, but later became well perfused with the administration of fraction of inspired oxygen (FIO2) 100% and application of warm pads. There was no need for resection of the bowel and an entire small bowel run was performed with adhesiolysis from duodenojejunal (DJ) flexure to ileocecal junction. Postoperatively, he was shifted to special care. His initial complaint was of ileus which later settled and was progressed to liquid diet on post-op day 4 and to soft diet on 6th day postoperatively.

**Fig. 3:**
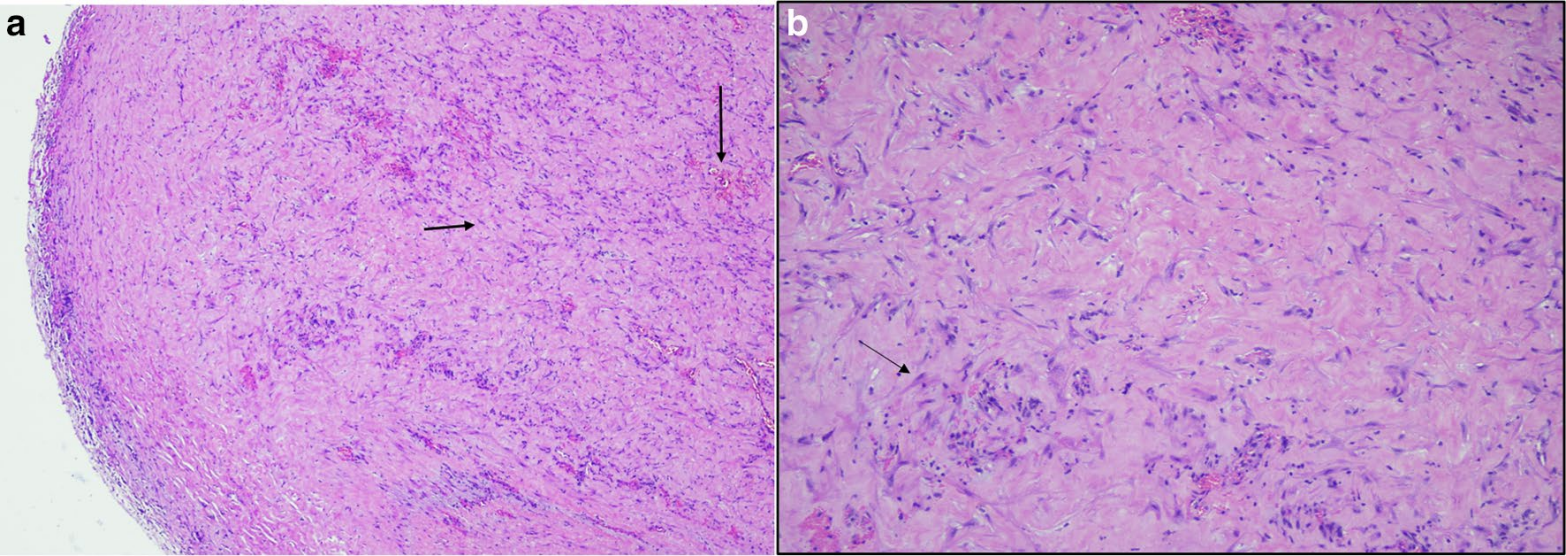
The figure demonstrates H&E staining showing increased cellularity, fibroblasts, inflammatory cells, and congested vessels. **a** Arrow (horizontal) fibroblast-like cells. Arrow (down) congested vessels. Original magnification ×40. **b** Original magnification ×100

He was discharged on the 6th day postoperatively on a soft diet and had normal bowel movements. He was followed up in the clinic on the 12th postoperative day and was doing well, tolerating a regular diet with normal bowel movements. His skin staples were removed and he was asked to follow-up after 3 months. Histopathology of the sclerotic tissue was reported as fibro-collagenous and adipose tissue exhibiting dense areas of hyalinization and fibrosis. There were scattered fibroblasts, mild inflammatory infiltrate comprising predominantly of lymphocytes and occasional plasma cells as well consisting of finding of sclerosing peritonitis or cocoon abdomen syndrome (Fig. 3a, b).

## Discussion

Abdomen cocoon syndrome was first described more than a century ago and was initially termed peritonitis chronica fibrosa incapsulata to describe the membrane encasing the intestine [5]. In most cases the cause of sclerosing peritonitis is unknown. However, patients on long-standing peritoneal dialysis, with peritoneal shunts, and those suffering from abdominal tuberculosis are more prone to develop this fibrous layer around the bowel [6]. The prevalence of abdominal cocoon syndrome (ACS) is unknown, but among peritoneal dialysis patients, the prevalence is 1.4–7.3% [7]. The female-to-male ratio is 1:1.35 and its incidence is more common in young females from tropical and subtropical countries mainly Pakistan, China, and India [8].

Patients usually present with intermittent attacks of partial bowel obstruction and with a history of receiving anti-tuberculosis therapy due to misdiagnosis. X-ray abdomen supine is non-specific while CECT shows entrapment of bowel loops inside the sclerosing layer forming a cocoon [9]. The role of laparoscopy is confined to elective presentations and aids in diagnosis, but management inadvertently requires open exploration because of the nature of adhesions and bowel entrapment [10]. Surgical treatment mandates excision of the entire sclerosing membrane resulting in entrapment of bowel loops and straightening all entrapped bowel loops to prevent future attacks of bowel obstruction. Resection and anastomosis are generally not required unless there is an iatrogenic injury of the bowel during adhesiolysis [11].

The histological findings in a case of sclerosing peritonitis show areas of increased cellularity comprising fibroblasts which in later stages may show hypocellularity. The matrix is composed of fibroconnective tissue. Besides, mononuclear inflammatory infiltrates, fibrin, and congestion of vessels are seen. The surface, if oriented may show denudation of the mesothelial layer. There should be no evidence of foreign body granulomas, giant cells, or the presence of any birefringent material [12].

Recent literature only consists of case reports and some case series and the majority of them are presented as chronic conditions [13]. This case report highlights a relatively unusual emergency presentation of sclerosing peritonitis with signs of focal peritonitis and sepsis. Cocoon syndrome as an emergency presentation with hemodynamic instability is extremely rare and treatment involves resuscitation and immediate exploration. As an emergency case, this condition can be misdiagnosed as either perforated viscus or internal herniation. However, treatment remains the same, which is excision of sclerosing membrane and straightening of the bowel. Series of cocoon abdomen cases as the emergency presentation will be more helpful to better understand the natural course of the disease.

## Conclusion

Cocoon abdomen is one of the rare causes of chronic bowel obstruction and its presentation with sepsis is extremely rare. In cases of emergency presentation, it is found at the time of laparotomy as an unexpected finding. Removal of the sclerosing membrane and straightening up of the bowel is the treatment of choice if the bowel on inspection is healthy and viable.

## Data Availability

All data will be available on request, keeping the anonymity of the patient.
